# Medical Service in the Navy

**Published:** 1859-01

**Authors:** 


### MEDICAL SERVICE IN THE NAVY.

From an article published in this number, under the head of "Medical Service in the Navy," it will be perceived that there is a large deficiency of Medical men in that "service," and if Congress should increase the Medical Corps in accordance with the recommendation of the Secretary of the Navy, it is probable that about fifty young gentlemen will be appointed to meet the demand of the service during the year 1859. Candidates for admission into the Medical Corps of the Navy, will therefore find this an exceedingly favorable opportunity for making application.

